# Recent innovations and challenges in the treatment of fungal infections

**DOI:** 10.3389/fcimb.2025.1676009

**Published:** 2025-10-01

**Authors:** Di Liu, Renjie Zhou, Xindi Gao

**Affiliations:** Department of Emergency, Xinqiao Hospital, Army Medical University, Chongqing, China

**Keywords:** fungal infections, antifungal drugs, combination therapy, biotechnology-driven approaches, drug development

## Abstract

The prevalence of fungal infections has been increasing consistently in recent years, particularly among immunocompromised individuals, resulting in increased mortality. The World Health Organization (WHO) now lists “super fungi”, such as *Candida auris* as global public health threats, highlighting the urgent requirement for new antifungal therapies. Although conventional agents such as azoles and polyenes remain prevalent in medical treatment, challenges including drug resistance, limited selectivity, and high toxicity limit their value, prompting the need for the development of more effective therapeutic strategies. Current research trends are shifting towards multi-mechanistic combination therapies and biotechnology-driven approaches, which demonstrate significant potential. This review summarizes recent advances and outlines directions for future antifungal drug development and new therapies.

## 1 Introduction

In recent years, the incidence of fungal infections has risen alarmingly, driven by immunosuppression from organ transplantation and chemotherapy for cancer treatment (Burki, 2023; Zhang et al., 2023; Ikuta et al., 2024). This population is significantly more likely to face severe infections and has high mortality rates (Pagano et al., 2017; Wiederhold and Gibas, 2018; Oberoi et al., 2023). Among the fungi of concern, *Candida auris* and *Aspergillus* have posed significant threats to patient safety and public health worldwide (Lamoth et al., 2017; Janniger and Kapila, 2021; Horton et al., 2023; Iliev et al., 2024; Proctor et al., 2025). The emergence of these resistant strains is exacerbated by the widespread use of antifungal medications, inadvertently contributing to the development of drug resistance (Fisher et al., 2022; Lockhart et al., 2023). This phenomenon poses a serious challenge to the management of fungal infections, and urgent and innovative therapeutic strategies are needed to address this growing public health crisis (Brown et al., 2012). Furthermore, fungal infections of the central nervous system (CNS), such as cryptococcal meningitis, represent a particularly devastating manifestation associated with high mortality rates (Ashley, 2019; Lino et al., 2024). Their management is compounded by the formidable challenge of the blood-brain barrier (BBB), which severely restricts the access of most antifungal drugs to the site of infection, necessitating prolonged treatment courses and highlighting an urgent need for novel therapeutic strategies (Nau et al., 2010; Schwartz et al., 2018).

Traditional antifungal therapies, including azoles, echinocandins, and polyenes, have been the keystone of treatment for these infections (Patterson, 2006; Saini et al., 2025). However, the limitations of these agents are becoming increasingly apparent, especially in the context of rapidly emerging resistance. For example, *Candida glabrata* and *C. auris* have developed significant resistance to multiple antifungal classes, complicating treatment regimens and leading to treatment failures. The challenge is further exacerbated by the poor bioavailability and pharmacokinetic profiles of many antifungal drugs, limiting their effectiveness in treating systemic infections (Lass-Florl, 2011; Lewis, 2011). Consequently, there is an urgent need for novel antifungal drugs with new mechanisms of action that are effective in targeting drug-resistant strains while minimizing toxicity to patients (Donlin and Meyers, 2022; Hoenigl et al., 2024).

In addition to the development of new antifungals, innovative strategies such as combination therapy and the use of nanotechnology for drug delivery are being explored to enhance the efficacy of existing treatments. Combination therapy, which involves using multiple antifungal agents concurrently, has shown promise in improving treatment outcomes and reducing the likelihood of resistance development (Toepfer et al., 2024; Wake et al., 2024). Furthermore, the integration of nanotechnology in antifungal drug delivery systems has the potential to enhance the bioavailability and targeted action of antifungal agents, thereby improving therapeutic outcomes. These advancements are crucial since they not only provide alternative treatment options but also open the way for a more personalized approach to the management of fungal infections in susceptible populations.

As the landscape of fungal infections is changing, the medical community must remain alert to monitor trends in antifungal resistance and adjust treatment strategies accordingly. Continued research into the mechanisms of drug resistance, the development of rapid diagnostic tools, and the exploration of novel therapeutic avenues are essential to address the challenges posed by invasive fungal infections. In face of this growing public health threat, the integration of multidisciplinary approaches, including microbiology, pharmacology, and immunology, will be crucial in developing effective interventions to safeguard patient health and improve outcomes.

## 2 Epidemiological status of fungal infections

The global burden of fungal infections is substantial, with millions of cases reported annually (Denning, 2024; Iliev et al., 2024). Recent estimates indicate that invasive fungal infections result in an annual incidence of 65 million invasive fungal infections and 3.8 million deaths (Denning, 2024) (**Table 1**). On October 25, 2022, WHO released its first fungal priority pathogen list, ranking 19 invasive fungi as critical, high, or medium priority based on incidence, mortality, resistance, and therapeutic options (Burki, 2023). The incidence of cryptococcal meningitis in HIV-positive individuals remains alarmingly high, with an estimated 223,000 cases and 181,000 deaths annually (Rajasingham et al., 2017). Additionally, the rise in antifungal resistance complicates treatment paradigms, with studies showing that an increasing number of *Candida albicans* isolates are resistant to commonly used antifungal drugs (Fisher et al., 2022; Sobel, 2023). These data underscore the urgent need for improved diagnostic, preventive, and therapeutic strategies to address the growing threat of fungal infections globally.

**Table 1:** Annual incidence and mortality of leading invasive fungal infections worldwide.

Type of infection	Annual incidence (estimated)	Annual deaths (estimated)	Mortality rate
Invasive Aspergillosis	2.1 million	1.8 million	85.20%
Chronic Pulmonary Aspergillosis	1.8 million	340,000	18.50%
Invasive Candidiasis	1.6 million	995,000	63.60%
Pneumocystis Pneumonia	505,000	214,000	42.40%
Cryptococcal Meningitis	194,000	147,000	75.80%
Other serious fungal infections	~300,000	~161,000	~53.7%

Fungal infections represent a significant public health challenge globally, characterized by complex epidemiological features influenced by various factors. Skin and nail infections are particularly common, and dermatophyte infections, caused primarily by *Trichophyton* spp., are the primary pathogens of skin, hair, and nail infections worldwide (Thakur, 2015; Chanyachailert et al., 2023). The epidemiology of these infections varies across different geographic regions and specific populations, and their prevalence patterns have changed over recent years (Havlickova et al., 2008; Coulibaly et al., 2018; Chanyachailert et al., 2023). Filamentous fungi also cause high mortality and pose a serious public-health threat. *Aspergillus*, *Mucorales* and *Fusarium* drive the high mortality of invasive fungal infections, especially in immunocompromised hosts, including patients with hematological malignancies, transplant recipients, and individuals with severe COVID-19 or liver disease (Barros et al., 2023; Harris, 2023; Hlaing et al., 2023; Oberoi et al., 2023).

The epidemiology of fungal infections is significantly influenced by the host’s immune status, particularly in immunocompromised individuals. Immunocompromise reduces the ability to generate an effective immune response, making these individuals more susceptible to opportunistic pathogens (Dobes et al., 2022; Chen et al., 2023). A multi-center study of 234 adult HSCT (Hematopoietic Stem Cell Transplantation) recipients found that invasive candidiasis comprised 24.8% of all invasive fungal infections (Neofytos et al., 2009). The use of immunosuppressive therapies further exacerbates this risk by impairing both innate and adaptive immune responses (Schleimer, 2005; Gregory et al., 2023).

Despite advances in antifungal therapy, mortality rates from cryptococcal meningitis remain unacceptably high, particularly in resource-limited settings and immunocompromised populations, underscoring its significance as a global health threat (Pyrgos et al., 2013). Cryptococcosis and aspergillosis are among the most common fungal infections affecting the CNS, often leading to severe morbidity and high mortality rates (Vu et al., 2014). The difficulty in treating these infections is compounded by the inefficiency of most antifungal drugs in penetrating the BBB, which significantly limits their therapeutic efficacy (Nau et al., 2010). Understanding the epidemiological trends and risk factors associated with fungal CNS infections is also crucial for developing targeted prevention and treatment strategies.

## 3 Classification, mechanism of action and limitations of traditional antifungal drugs

**Figure 1:**
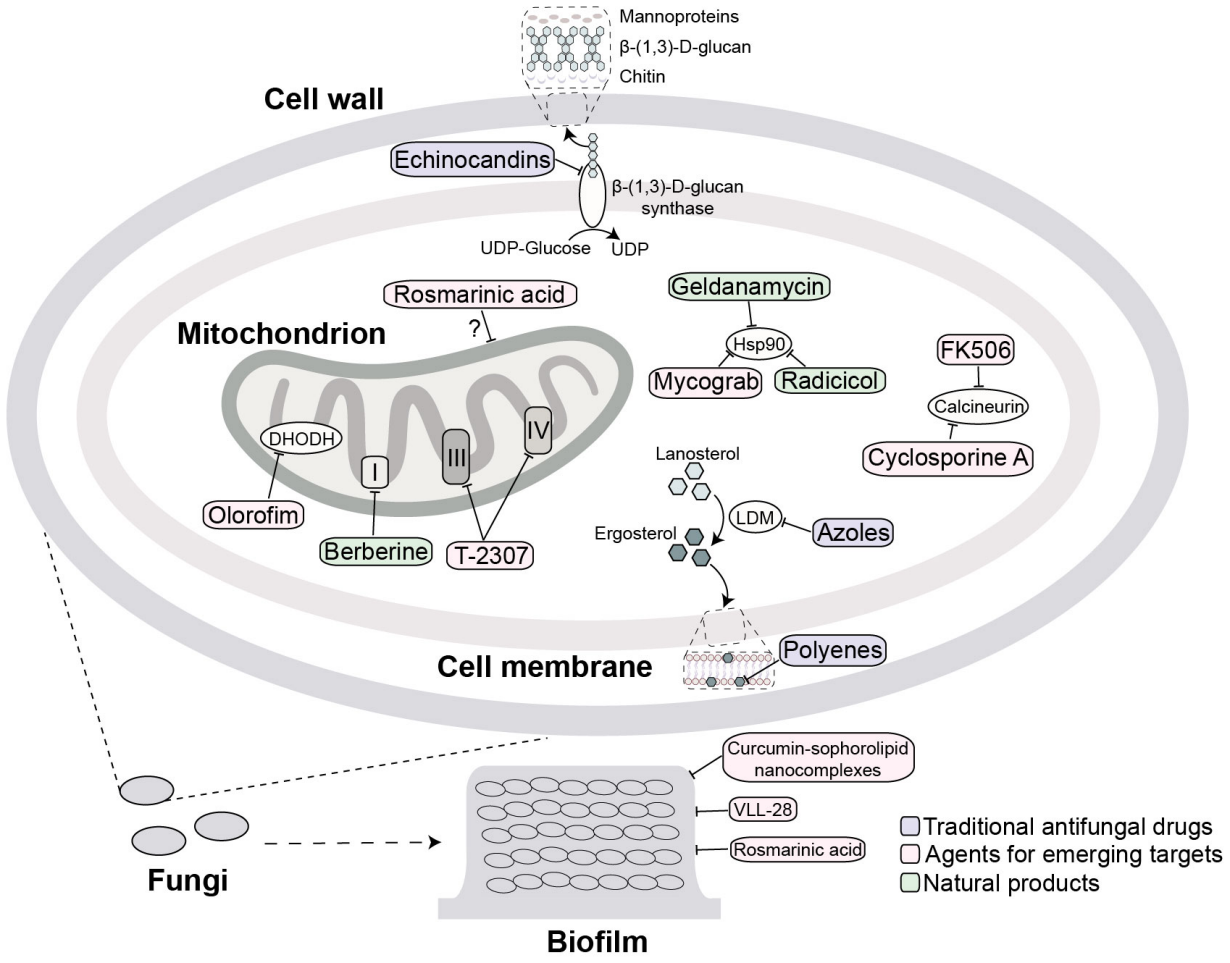
Antifungal agents targets and their mechanism of action. Traditional key targets include: surface β-(1,3)-D-glucan synthase (inhibited by echinocandins); ergosterol (its biosynthesis inhibited at lanosterol demethylase (LDM) by azoles or directly bound by polyenes). Emerging targets include: mitochondria (target by rosmarinic acid, berberine, T2307 and olorofim); calcineurin (targeted by cyclosporine A and FK506); and Hsp90 (targeted by geldanamycin, radicicol and Mycograb). Extracellular biofilm disruption is targeted by agents such as curcumin-sophorolipid nanocomplexes, VLL-28, and rosmarinic acid. The purple, pink and green squares mark traditional antifungal drugs, agents for emerging targets and natural products, respectively.

Invasive fungal infections caused by *Candida*, *Aspergillus*, and *Cryptococcus* are now major public health concerns. The mainstay of therapy remains three drug classes-azoles, polyenes, and echinocandins (**Figure 1**), but the effectiveness of these drugs is declining due to their toxicity, drug-drug interactions and rapidly expanding resistance (Berman and Krysan, 2020).

### 3.1 Azole antifungal agent

Azoles represent a widely utilized class of antifungal agents, with numerous compounds developed over the years and ongoing efforts to introduce new derivatives (Kim et al., 2022). Their mechanism of action involves the inhibition of lanosterol 14-α-demethylase (LDM), a key enzyme contributing to the 14-α-demethylation of lanosterol, thereby blocking the ergosterol biosynthesis pathway. This inhibition disrupts the integrity of the fungal cell membrane by preventing ergosterol production and promoting the accumulation of toxic sterol intermediates, ultimately impairing fungal growth and leading to cell death (Maertens, 2004).

Azole antifungals, such as fluconazole and itraconazole, are now widely used for the treatment of various fungal infections due to their efficacy and oral bioavailability. However, their clinical utility has been hampered by several challenges. One of the most significant issues is the emergence of drug resistance in fungal pathogens, particularly *C. albicans* and *Aspergillus fumigatus*. Resistance mechanisms include alterations in drug target, overexpression of efflux pumps, and biofilm formation, which significantly reduce the efficacy of azole therapy (Kaur and Nobile, 2023; van de Veerdonk et al., 2025). Besides resistance, dose-limiting toxicity is another major obstacle to prolonged azole use. The most common is dose-dependent hepatotoxicity, which can range from asymptomatic transaminase elevation to acute liver injury (Song and Deresinski, 2005; David and Hamilton, 2010; Buhler et al., 2019). Moreover, certain azoles like voriconazole and fluconazole can increase the risk of serious cardiac arrhythmias (Gueta et al., 2017; Berger et al., 2018; Kitaya et al., 2024). Another pivotal toxicity is endocrine disruption, most pronounced with ketoconazole, which potently inhibits steroidogenesis and may precipitate adrenal insufficiency and gynecomastia (Benitez and Carver, 2019). Additionally, azole drugs have significant drug-drug interactions, especially in immunocompromised patients who are often on multiple medications. These interactions can lead to increased toxicity or reduced efficacy of either the azole or the co-administered drugs (Patterson et al., 2016). Furthermore, the pharmacokinetics of azoles can vary among individuals due to factors such as genetic polymorphisms in drug-metabolizing enzymes, which complicate treatment regimens (Weiss et al., 2009; Lee et al., 2012).

### 3.2 Polyene drugs

Amphotericin B (AmB), discovered in 1956, remains the gold standard for systemic antifungal therapy because of its broad spectrum and low resistance rate (Sundriyal et al., 2006). Among polyenes, AmB, nystatin, and natamycin are the most widely utilized (Carolus et al., 2020; Carmo et al., 2023). AmB exerts its antifungal effect by binding to ergosterol, an essential component of the fungal cell membrane, thereby forming transmembrane pores that disrupt membrane integrity and induce intracellular damage (Carolus et al., 2020). Given its poor oral bioavailability, AmB is administered intravenously and often used in combination with other antifungal agents, such as flucytosine or fluconazole for the treatment of severe *Cryptococcus* and *Candida* infections (Boutin and Luong, 2024), or with echinocandins or azoles for aspergillosis and mucormycosis (Carmo et al., 2023). Nephrotoxicity is the principal limitation and can lead to renal failure, especially in vulnerable patients (Gursoy et al., 2021). The high intrinsic toxicity of AmB has prompted the development of lipid formulations aimed at reducing side effects while maintaining antifungal efficacy (Wingard et al., 2000), yet adverse effects and the need for renal monitoring still restrict use. Moreover, the emergence of resistant fungal strains further complicates the therapeutic landscape, as some species exhibit reduced susceptibility to polyenes (Young et al., 2003; Vandeputte et al., 2007, 2008; Purkait et al., 2012; Ponte-Sucre et al., 2017). The development of resistance mechanisms, including alterations in ergosterol biosynthesis, poses a significant challenge to the effective use of polyene antifungals (Vandeputte et al., 2008).

### 3.3 Echinocandins

Echinocandins comprise important antifungal agents, such as caspofungin, micafungin, and anidulafungin, which are approved for clinical use and exert their antifungal effects by inhibiting the synthesis of β-(1,3)-D-glucan in the fungal cell wall (Misiek and Hoffmeister, 2007; Szymanski et al., 2022). Given the high abundance of glucans in the fungal cell wall and their absence in mammalian cells, this mechanism of action confers strong antifungal efficacy with minimal host toxicity. Clinically, echinocandins serve as first-line therapy for candidiasis and are combined with other antifungals for aspergillosis (Mroczynska and Brillowska-Dabrowska, 2020). Echinocandins exhibit water solubility and are formulated as lyophilized powders that must be administered intravenously due to their low oral bioavailability and poor gastrointestinal absorption (Szymanski et al., 2022). Adverse events are less common than with older agents but can include edema, bronchospasm, dyspnea, and hypotension (Szymanski et al., 2022). However, the emergence of resistance to echinocandins poses a significant challenge in clinical practice. The primary mechanism of echinocandin resistance involves mutations in the *FKS* genes, which encode the catalytic subunit of β-(1,3)-D-glucan synthase, the target of echinocandins. These mutations occur in specific “hot spot” regions and reduce the enzyme’s sensitivity to the drug, leading to higher minimum inhibitory concentrations (MIC) (Wiederhold, 2016; Hori and Shibuya, 2018).

The historical development of fungal infection treatment has been a complex and lengthy process, involving drug research and development, advances in diagnostic technology, and continuous optimization of clinical practice. Early research on fungal diseases began in the late 19th century, including the discovery of histoplasmosis and other conditions. During this period, medical understanding of fungal diseases remained in its exploratory phase, lacking systematic treatment protocols (Espinel-Ingroff, 1996). In 1979, studies demonstrated that AmB combined with flucytosine, a drug which inhibits the synthesis of DNA, was more effective than monotherapy in treating cryptococcal meningitis (Bennett et al., 1979). In 1992, fluconazole proved superior to AmB in preventing recurrence of cryptococcal meningitis. In 2004, the treatment of systemic fungal infections involved the use of multiple antifungal agents, such as polyenes, imidazole, and triazoles (Wu et al., 2004). However, the treatment of fungal infections continues to face challenges, such as resistance to antifungal drugs, the complexity of drug development, and limitations in diagnostic technologies.

### 3.4 Natural products

Natural products have long been recognized for their therapeutic potential, and their application in antifungal therapy is gaining fresh interest due to the increasing incidence of drug-resistant fungal infections (Soares et al., 2013; Dong et al., 2023). These compounds, derived from various sources such as plants, fungi, and marine organisms, exhibit a wide range of bioactivity that can be harnessed for antifungal applications.

Essential oils are volatile blends dominated by mono- and sesquiterpenes that preserve food but are seldom used as antifungals (Bakkali et al., 2008). They permeabilize membranes, vacuolate cytoplasm, and disorganize hyphae, primarily acting fungistatically but causing lysis at high dose (Basak and Guha, 2018). Eugenol, a phenol that reduced ergosterol synthesis, and its derivatives can inhibit the growth of *C. albicans* and *Candida parapsilosis* with MIC ranging from 50 to 100 μg mL^-1^, and showed a similar binding pattern of fluconazole and posaconazole at the LDM binding site (Dutra et al., 2023). Additionally, the hydroalcoholic plant extract of fresh leaves of *Aloe vera* is effective in inhibiting the mycelial growth of multiple pathogenic fungi, such as *Botrytis gladiolorum* and *Fusarium oxysporum* (Rosca-Casian et al., 2007). Black pepper oil, a natural preservative with antioxidant, hepatoprotective and antifungal activities, can alter the membrane permeability in *Aspergillus flavus*, *Aspergillus ochraceus*, *Fusarium graminearum* and *Penicillium viridans*, leading to mitochondrial dysfunction. The composition of this oil is complex, rich in mono- and sesquiterpenes, as well as phenols, flavonoids, and proanthocyanidins, and the nature of the bioactive molecules involved in the antifungal action is not known (Zhang et al., 2021). Propolis, a resinous hive product abundant in polyphenolic flavonoids and caffeic acid derivatives, has emerged as a promising adjuvant in anti-cryptococcal therapy. Propolis from the stingless bee significantly reduces cell wall-associated melanin in *Cryptococcus neoformans*, thereby enhancing macrophage-mediated fungal clearance without detectable cytotoxicity (Mamoon et al., 2020). Moreover, Argüelles et al. showed that a 1:4 combination of propolis and carnosic acid exhibited a synergistic fungicidal effect on *C. neoformans* without causing detectable changes in cellular morphology. Furthermore, exposure of mature biofilms to various carnosic acid–propolis formulations diminished the metabolic activity of the sessile cells forming biofilms (Arguelles et al., 2021).

Recent research has identified numerous natural products with significant antifungal properties, which have shown efficacy against common fungal pathogens like *Candida*, *Aspergillus*, or *Cryptococcus* spp (Dong et al., 2023). The mechanisms of action of these natural antifungals often involve disrupting fungal cell membranes and inhibiting cell wall synthesis (Curto et al., 2021; Kowalczyk, 2024), making them valuable candidates for drug development. Studies have shown that potent antifungal natural products in the structural classes such as flavonoids, terpenoids, and alkaloids have significant efficacy against *C. albicans* and *Aspergillus* spp. Their mechanisms involve targeted disruption of fungal membrane integrity via ergosterol complexation, inhibition of β-glucan synthase, or interference with essential metabolic pathways like chitin biosynthesis (Sultan and Ali, 2011; Wijnants et al., 2022; Jayaweera et al., 2025).

The integration of natural products into antifungal therapy not only provides a sustainable alternative to synthetic drugs, but also opens the way for the discovery of novel compounds that can be used in combination therapies to combat drug-resistant infections effectively. Substantial evidence confirms synergistic interactions between clinical antifungals and natural products, including essential botanical oils (*Mentha, Pelargonium, Allium, Origanum, and Thymus* spp.) (Bhattacharya et al., 2021) and purified phytochemicals (berberine, curcumin, cinnamyl alcohol, eugenol, magnolol, allicin) (Herman and Herman, 2023). Berberine chloride, found in many plants and acting on several enzymatic activities, can enhance the fungistatic activity of fluconazole against fluconazole-resistant clinical isolates of *C. albicans* (Quan et al., 2006). Augostine et al. screened 800 natural-product pairs and identified 34 synergistic combinations containing eugenol, β-escin, curcumin, or berberine; three of these combinations inhibited growth of human and plant pathogens, including *C. albicans*, *A. fumigatus*, *Zymoseptoria tritici* and *Botrytis cinerea*, and overcame azole resistance and biofilms (Augostine and Avery, 2022). Moreover, although carnosic acid and propolis polyphenols alone display modest antifungal potency (Ota et al., 2001; Birtic et al., 2015), their combination can act synergistically against *C. neoformans* (Arguelles et al., 2021) and *C. albicans* (Arguelles et al., 2020).

However, the effectiveness of these traditional antifungal drugs in treating CNS infections is significantly hindered by their poor penetration of the BBB. The BBB is a protective structure that regulates the movement of particles into and out of the brain, making it difficult for many drugs to reach therapeutic concentrations in the CNS (Reguera-Gomez et al., 2023). Based on published physicochemical and pharmacokinetic data, Wirth et al. systematically summarized the ability of antifungal drugs to penetrate the CNS. Although AmB achieves low concentration in cerebrospinal fluid, it remains the most effective drug for treating fungal CNS infections (Wirth and Ishida, 2020). Studies have investigated whether there is a correlation between the MICs of AmB and treatment efficacy (Andes, 2006; Park et al., 2006). However, current evidence indicates that the relationship between AmB concentration in cerebrospinal fluid and the therapeutic outcome of CNS fungal infections is not obviously significant (Kethireddy and Andes, 2007). The concentrations of AmB in cerebrospinal fluid during meningitis progression in both animals and humans remain below 1% (Wirth and Ishida, 2020). This may be attributed to the presence of CNS lesions causing BBB disruption, thereby enhancing the permeability of antifungal agents (Pyrgos et al., 2010). Additionally, evidence suggests that fungal cell wall components such as *Cryptococcus* glucuronoxylomannan (GXM) can weaken the BBB by modulating the RhoA pathway in brain microvascular endothelial cells (Lee et al., 2023; Reguera-Gomez et al., 2023). Therefore, the combination of drugs targeting this pathway with traditional antifungal agents may enhance the efficacy of treating CNS infections.

## 4 The development strategies of antifungal agents and medical therapy

### 4.1 Discovery and validation of emerging targets

Traditional antifungal agents used for the treatment of systemic infections primarily target three key fungal structures: ergosterol synthesis (azoles), ergosterol directly (polyenes), or cell wall synthesis (echinocandins). Recent advances have substantially expanded our understanding of structural and functional components of fungal pathogens, clarifying how these pathogens establish infection (Odds et al., 2003; Perfect, 2017). Developing new antifungal agents and strategies is crucial, especially for CNS infections where the BBB limits drug penetration (Reguera-Gomez et al., 2023). Current research focuses on identifying new antifungal targets, combining antifungal drugs with complementary mechanisms of action, and developing advanced delivery systems, such as nanoparticles and liposomes, thereby enhancing drug bioavailability and therapeutic efficacy at fungal infection sites. This section reviews emerging antifungal targets and the mechanism-based compounds moving through preclinical development (**Figure 1**).

#### 4.1.1 Calcineurin

The calcineurin signaling complex represents an established therapeutic target for novel antifungal development, given its validated regulatory functions in fungal growth, stress adaptation, and virulence expression across diverse pathogenic species (Rusnak and Mertz, 2000; Park et al., 2016; Juvvadi et al., 2017). Calcineurin constitutes a dimeric serine/threonine-specific protein phosphatase complex, composed of a regulatory B subunit [calcineurin B (CnB)] and catalytic subunit [calcineurin A (CnA) (Rusnak and Mertz, 2000), activated by Ca²^+^-calmodulin signaling and exhibiting high structural and functional conservation across eukaryotic organisms. Calcineurin orchestrates distinct regulatory functions across biological systems: in mammalian T cells, it modulates transcription factors such as nuclear factor of activated T cells to control interleukin-2-mediated immune activation (Steinbach et al., 2007). Conversely, in pathogenic fungi, this phosphatase serves as a central node for stress response pathways governing growth, antifungal resistance, and virulence (Rusnak and Mertz, 2000; Steinbach et al., 2007; Park et al., 2016). Therapeutically, calcineurin is inhibited by immunosuppressants tacrolimus FK506 and cyclosporine A (CsA), which exhibit broad-spectrum activity against *C. neoformans*, *Candida* spp., and *A. fumigatus* (Odom et al., 1997b; Blankenship et al., 2003; Steinbach et al., 2006). However, their utility as antifungal agents is precluded due to concurrent host immunosuppression. Current strategy to circumvent this limitation is the development of FK506 analogues that selectively penetrate fungal membranes while inert in mammalian systems (Nambu et al., 2017). However, the translational potential of FK506 and CsA for antifungal drug development remains constrained by dose-limiting immunosuppressive effect. Analogues of FK506 by biosynthesis or pharmacological modification exhibited reduced immunosuppressive activity but maintained significant antifungal activity (Odom et al., 1997a; Beom et al., 2019; Gobeil et al., 2021; Hoy et al., 2022). Recent elucidation of the crystallographic structures of CnA and CnB subunits in complex with FK506 and its cognate binding protein FKBP12 in *A. fumigatus*, *C. albicans*, and *C. neoformans* provides a structural foundation for rational design of novel FK506 analogs with enhanced antifungal specificity (Juvvadi et al., 2019; Rivera et al., 2023).

#### 4.1.2 Hsp90

Hsp90 represents an evolutionarily conserved heat shock protein that couples the free energy derived from ATP hydrolysis to facilitate the folding and conformational stabilization of conformationally labile client proteins, including kinases, transcription factors, and signal transduction regulators (Richter et al., 2010). The therapeutic appeal of targeting Hsp90 arises from its role as a central signaling hub that regulates growth, stress adaptation, antifungal resistance, and virulence across diverse fungal pathogens (Cowen and Lindquist, 2005; Shapiro et al., 2009; Singh et al., 2009; Taipale et al., 2010; Leach et al., 2012; Robbins et al., 2012; Lamoth et al., 2016).

Natural products geldanamycin and radicicol are inhibitors of Hsp90, replacing ATP and blocking the function of Hsp90 (DeBoer et al., 1970; Schulte et al., 1998). Hsp90 inhibitors potentiate fluconazole efficacy against *C. albicans* infection in a *Galleria mellonella* model (Cowen et al., 2009). However, the high conserved characteristic of Hsp90 results in toxicity when inhibited, thus limiting the utility of contemporary Hsp90 inhibitors in mammalian fungal infection models (Taipale et al., 2010). A recombinant 28kDa monoclonal antibody that binds to fungal Hsp90, Mycograb (efungumab [Mycograb; NeuTec Pharma/Novartis]) was developed. Both *in vitro* and animal studies revealed that Mycograb in combination with AmB has a synergistic effect on a variety of pathogenic *Candida* spp (Matthews et al., 2003). In addition, better clinical validation was shown in patients with invasive candidiasis who were given Mycograb antibody in combination with AmB (Pachl et al., 2006). Notably, marketing authorization for Mycograb was withheld by regulatory authorities in November 2006 owing to quality concerns. Subsequently, a modified variant designated Mycograb C28Y underwent development; however, this reformulated therapeutic formulation demonstrated reduced efficacy compared to the original antibody formulation (Bugli et al., 2013).

#### 4.1.3 Mitochondria

Mitochondria serve as the primary ATP-generating organelles in eukaryotes, driving oxidative phosphorylation and tricarboxylic acid cycle to produce cellular ATP. Beyond energy metabolism, fungal mitochondria regulate amino acid synthesis, phospholipid biogenesis, virulence, and antifungal resistance through respiration-coupled processes (Chatre and Ricchetti, 2014; Chang and Doering, 2018; Malina et al., 2018; Li et al., 2020), thus it has emerged as an antifungal target.

Rosmarinic acid significantly directly inhibited *C. albicans* mitochondrial function, with MTT assay data indicating over 50% reduction in metabolic activity (Ivanov et al., 2022). Berberine accumulates in fungal mitochondria, impairing membrane potential and inhibiting Complex I activity. This compound also disrupts Mdr1p-mediated efflux in *C. albicans*, reversing multidrug resistance. *In vivo* studies confirm the efficacy of this compound by prolonging survival in murine models of disseminated Mdr1p-overexpressing candidiasis (Tong et al., 2021).

The arylamidine compound T-2307 demonstrates broad-spectrum fungicidal activity against *Candida*, *Aspergillus*, and *Cryptococcus* spp. *in vitro* and prevents disseminated murine infections (Nishikawa et al., 2017; Wiederhold et al., 2020; Gerlach et al., 2021). Notably, polyamine transporters are minimally expressed in mammalian cells such as rat hepatocytes (Nishikawa et al., 2010, 2016), and targeted inhibition of mitochondrial Complex III and IV through selective uptake of fungal polyamine transporters can lead to membrane potential collapse of fungal mitochondria (Shibata et al., 2012; Yamashita et al., 2019). The negligible effect on rat mitochondria highlights its therapeutic selectivity (Shibata et al., 2012). ATI-2307 is currently in advanced preclinical development (licensed to Appili Therapeutics in 2019) and has demonstrated a favorable safety profile in Phase I clinical trials.

Olorofim (F901318) is a first-in-class orotomide antifungal that selectively inhibits fungal dihydroorotate dehydrogenase (DHODH), a mitochondrial enzyme critical for *de novo* pyrimidine synthesis, with a selectivity 2,000-fold greater than that of the human enzyme (Oliver et al., 2016). It effectively disrupts DNA/RNA synthesis and virulence in molds and dimorphic fungi but is inactive against yeasts like *Candida* and *Cryptococcus* (Buil et al., 2017). In murine models of invasive aspergillosis, Olorofim improved survival by inhibiting fungal growth (Oliver et al., 2016; du Pre et al., 2018). Olorofim is currently in the global Phase III clinical trial (OASIS study, NCT05101187), aiming to evaluate its efficacy and safety in patients with invasive aspergillosis.

#### 4.1.4 Biofilms

Fungal biofilms, structured communities embedded in extracellular matrix, are emerging therapeutic targets. Their dense sterol-rich layers, persister cells, up-regulated efflux pumps, and drug-restricting polysaccharide-protein matrix enable immune evasion and multifactorial resistance (Kernien et al., 2017; Pierce et al., 2017; Silva et al., 2017). Innovative anti-biofilm strategies are exemplified by: (1) curcumin-sophorolipid nanocomplexes disrupting *C. albicans* biofilm structure through downregulation of hyphal regulators (*SAP4, HWP1, HYR1*) and resistance genes (*ERG11*) (Rajasekar et al., 2021); (2) archaeal-derived antimicrobial peptide VLL-28 damaging cell wall integrity (Roscetto et al., 2018); (3) rosmarinic acid suppressing exopolysaccharide production and biofilm maturation (Ivanov et al., 2022) and (4) photodynamic therapy eradicating *C. auris* biofilms via photosensitizer-activated reactive oxygen species (ROS) (Bapat and Nobile, 2021). These approaches target previously inaccessible resistance mechanisms, such as matrix disruption, morphogenetic interference, and physical ablation, while highlighting the urgent need for agents with both biofilm disruption and immunomodulatory effects to overcome therapeutic barriers.

Target-driven antifungal drug development is a promising strategy to address the growing burden of fungal infections and the rise of antifungal resistance. By focusing on specific molecular targets, this approach offers the potential for more precise, effective, and safer therapies. However, overcoming the existing challenges will require sustained investment, innovation, and collaboration across the scientific community.

### 4.2 Combination therapy

Multi-mechanism combination therapy addresses core limitations of conventional antifungals, particularly drug resistance and pharmacokinetic constraint, by harnessing synergistic interactions between agents targeting distinct pathogenic pathways. This approach, validated through quantitative frameworks like the fractional inhibitory concentration index (FICI<0.5 defining synergy), builds upon antibacterial combination principles now adapted to medical mycology (Ganesan et al., 2004; Shaban et al., 2020; Carnero et al., 2025).

Cryptococcal infections, particularly cryptococcal meningitis, remain a significant global health challenge, especially among immunocompromised individuals. The limited antifungal drugs and the emergence of resistance have necessitated the development of multi-mechanism combination therapies to improve treatment outcomes. Combination therapy targets cryptococcal infections through multiple mechanisms: AmB: Disrupts fungal cell membranes by binding to ergosterol, leading to cell lysis. Flucytosine: Inhibits DNA and RNA synthesis by converting to 5-fluorouracil within fungal cells. Fluconazole: Inhibits ergosterol synthesis, weakening the fungal cell wall and enhancing the effects of other antifungals. This multi-mechanism approach not only improves fungal clearance but also reduces the likelihood of resistance development (Day et al., 2013).

The combination of AmB and flucytosine is considered the gold standard for treating cryptococcal meningitis. In clinical studies, this combination was effective against around 37% of isolates (Bandalizadeh et al., 2020). Another effective combination is AmB with fluconazole. Fluconazole inhibits ergosterol synthesis, complementing the membrane-disrupting action of AmB. This combination has shown synergistic effects in 31% of cases, making it a viable alternative, especially in regions where flucytosine is unavailable. Additionally, the combination of fluconazole plus flucytosine has demonstrated synergy in 12.5% of isolates, providing another therapeutic option (Bandalizadeh et al., 2020). The combination of fluconazole and terbinafine is also effective. Terbinafine inhibits squalene epoxidase and this combination has demonstrated significant correlations in minimum inhibitory concentrations for both species (Taha et al., 2024). Recent studies have demonstrated that One-week AmB combined with flucytosine, and two-week fluconazole combined with flucytosine regimens are effective as induction therapy for cryptococcal meningitis in resource-poor areas. Meanwhile, side effects (e.g., severe anemia) are more likely to occur with the two-week regimen than with the one-week AmB (Molloy et al., 2018). Additionally, single high-dose liposomal AmB combined with flucytosine and fluconazole has shown promise, with efficacy comparable to standard therapy while reducing adverse events (Jarvis et al., 2022).

Clinical drugs available for the treatment of candidiasis are confined to four mechanistic classes: polyenes, azoles, allylamines, and echinocandins. Compounds such as azoles and echinocandins inhibit essential *C. albicans* pathways, resulting in the rapid development of drug-resistant mutants; in contrast, AmB retains a lower incidence of resistance. Zhu et al. found that *Artemisinin* (ART), antimalarial active molecules, can act as a potentiator of AmB, effectively inhibiting *C. albicans* colonization and oral candidiasis (Zhu et al., 2021). ART upregulates ergosterol biosynthesis genes (*ERG1, ERG3, ERG9, ERG11*) and raises membrane ergosterol in *C. albicans*, thereby enhancing AmB binding and sensitivity (Zhu et al., 2021). Besifloxacin, an FDA-approved antibacterial, also shows antifungal activity. Combined with fluconazole, it reduced the MIC of fluconazole from 2 to 0.5 mg L^-1^ and, at 100 mg/kg/day, lowered renal fungal burden by 83% in a murine systemic candidiasis model (Chakraborty et al., 2025).

Panobinostat, an FDA-approved pan-histone deacetylase inhibitor, synergizes with fluconazole against azole-resistant *C. albicans*, reducing the MIC from 128 to 0.5–2 μg mL^-1^ and that of fluconazole from >512 to 0.25–0.5 μg mL^-1^. Panobinostat combined with fluconazole is synergistic against both planktonic *C. albicans* and its biofilms and markedly improves survival in *Galleria mellonella* infection model (Su et al., 2020). Chai et al. showed that quinoline-chalcone derivatives combined with fluconazole suppress azole-resistant *C. albicans* growth and biofilm formation, trigger ROS accumulation, compromise mitochondrial integrity, and deplete intracellular ATP (Chai et al., 2023).

Combination therapy represents a promising approach to overcoming the challenges posed by antifungal resistance and drug toxicity. By utilizing the synergistic effects of multiple agents, this strategy not only improves treatment efficacy but also reduces the risk of resistance and adverse effects. As fungal infections rise, combination therapy will become essential for better patient outcomes and for advancing antifungal strategies.

### 4.3 Biotech-driven antifungal drug development

Biotechnology-driven approaches are important for drug target discovery and identification and optimization of novel antifungal compounds with unique mechanisms of action. For example, the application of CRISPR-Cas9 technology has facilitated the exploration of fungal genetics, allowing researchers to create targeted mutations that can reveal potential drug targets and pathways involved in antifungal resistance (Hassane et al., 2025). Scientists have developed CRISPR-Cas9 genome editing system for many filamentous fungi, including *Trichoderma reesei*, *Neurospora crassa* and *Aspergillus nidulans* (Matsu-Ura et al., 2015; Nodvig et al., 2015; Al Abdallah et al., 2017). Prior to CRISPR-Cas9, fungal genome editing relied on homologous recombination and ZFNs (Zinc Finger Nucleases), which were labor-intensive and inefficient, but CRISPR-Cas9 overcame these limitations, permitting simultaneous, high-efficiency multiplex gene editing (Arnau et al., 1991; Zhang et al., 2024). CRISPR-Cas9-mediated fungal mutagenesis, as exemplified by *FKS1* editing in *C. glabrata* for echinocandin target validation, has been employed to identify resistance mechanisms (Hou et al., 2019). In addition, CRISPR-Cas9 has been used in *Fusarium graminearum*, the wheat scab pathogen, to inactivate mycotoxin biosynthetic genes, thereby attenuating virulence (Luo et al., 2023). Targeted genome editing can also attenuate fungal virulence, enabling the construction of avirulent strains (Song et al., 2019).

Moreover, nanotechnology-based formulations improve the solubility, delivery, and efficacy of both existing and new antifungal agents, addressing issues related to solubility and bioavailability and making optimized dosing feasible. Using an *in vitro* fungal-infected epithelial model, Lestner et al. characterized the pharmacokinetics and pharmacodynamics of different AmB formulations, indicating the importance of optimizing drug absorption for disease treatment (Lestner et al., 2010). Lipid nanocapsules (LNCs) have emerged as a multifunctional and efficient drug delivery platform, particularly for hydrophobic and challenging drugs like AmB. LNCs are prepared using solvent-free, low-energy methods such as the phase inversion temperature process, which is scalable and energy-efficient. This makes the production of AmB-loaded LNCs feasible for industrial applications (Aparicio-Blanco et al., 2019; Urimi et al., 2021). LNCs reconstituting AmB (Nano-LAmB) enhance cerebral spinal fluid penetration by 8-fold in cryptococcal meningitis models (Gazzoni et al., 2012; Alvarez-Uria et al., 2018).

Improvement and optimization of currently available antifungals also play a crucial role in the treatment of pathogenic fungi. AmB, a natural bacterial polyene, serves as the ultimate therapeutic option for systemic mycoses. Its potent fungicidal activity is counterbalanced by dose-limiting nephrotoxicity, confining its use to refractory infections. Maji et al. have elucidated the structural basis of the membrane activity of AmB using a modular synthetic platform guided by crystallographic, functional and in silico analyses. Rationalization of its ergosterol- and cholesterol-binding structural domains yielded an analog that selectively extracts fungal ergosterol while retaining mammalian cholesterol, thereby preserving glomerular integrity. This improvement is expected to enhance the efficacy and safety of treating life-threatening fungal diseases (Maji et al., 2023). In another recent study, Deng et al. utilized a phylogenetically-guided discovery strategy to target polyene macrolides containing an aminodeoxyglucose mycosamine and identified a promising antifungal candidate drug mandimycin, which exhibits potent, broad-spectrum fungicidal activity *in vitro* and *in vivo* against multidrug-resistant isolates (Deng et al., 2025). Rather than binding ergosterol, mandimycin disrupts membrane phospholipids and triggers ion efflux. Therefore, it remains effective against drug-resistant strains that target ergosterol, while being more soluble and less nephrotoxic (Deng et al., 2025).

Biotechnology has enabled the efficient production of antifungal peptides (AMPs). AMPs have emerged as promising candidates due to their broad-spectrum activity and low propensity for resistance development. Recent studies have highlighted the efficacy of antifungal peptides and lipopeptides, which exhibit potent activity against various fungal pathogens, including drug-resistant strains (Neubauer et al., 2020; Dos Reis et al., 2023; Helmy and Parang, 2023; Zhou et al., 2024). PAF102 peptide, designed for enhanced antifungal activity, has been successfully produced in *Pichia pastoris* using oleosin fusion technology. This method allows high yields of up to 180 mg L^-1^ of yeast culture, with the peptide accumulating in lipid droplets for easy extraction (Popa et al., 2019). Proteus mirabilis urease β subunit (PmUreβ) has demonstrated significant antifungal activity against *Candida* spp., reducing viability by over 50% at low concentrations, with potency varying by species and incubation temperature. PmUreβ further reduced *C. albicans* biofilm formation and its antifungal activity arises from disruption of cell-wall integrity without damaging the cell membrane, making it a potential alternative to conventional antifungals (Perin et al., 2025). The antifungal protein, naturally secreted by the filamentous ascomycete *Aspergillus giganteus*, exhibits potent activity against human and phytopathogenic fungi without affecting the viability of bacteria, yeast, plant and mammalian cells (Meyer, 2008).

Biotechnology is reshaping antifungal drug development by offering new tools to overcome the shortcomings of current therapies. Nanomaterials, genetic engineering, and the improvement of drug structures are already expanding treatment options. However, current biotechnology-driven approaches face multiple risks in clinical applications. Primary concerns regarding gene editing technologies include off-target effects, which may lead to genotoxicity and disruption of essential cellular processes; the largely unexplored long-term ecological implications of releasing genetically modified organisms; and the technical difficulty of achieving efficient and safe *in vivo* delivery of editing machinery to specific fungal cells (Krappmann, 2017). AMPs face limitations such as proteolytic degradation, unfavorable pharmacokinetics, and potential nephrotoxicity at higher doses. A critical safety concern is their propensity to induce hemolysis and cytotoxicity in host cells (Biswaro et al., 2018). Moreover, despite their initial high efficacy, the long-term use of AMPs may ultimately drive resistance development (Pimienta et al., 2022). For nanoparticle-based delivery systems, the intrinsic toxicity profiles of the carriers require thorough investigation (Biswaro et al., 2018). Therefore, further studies are needed to optimize the effectiveness, safety and scalability of these technologies.

## 5 Conclusion

Fungal infections by WHO-critical priority pathogens impose significant mortality burdens globally. While traditional antifungals possess well-defined mechanisms, their clinical application remains hampered by increasing drug resistance. Therefore, the development of new therapeutic approaches is particularly important. Beyond calcineurin, Hsp90, and mitochondrial pathways, fundamental research must uncover new targets to sustain antifungal drug discovery. Drug combinations offer possibilities for the treatment of fungal diseases, including combinations of classical antibiotics with other natural products such as ART. Meanwhile, biotechnology is accelerating antifungal innovation: CRISPR-Cas9 rapidly identifies essential genes related to pathogenicity and drug-resistance; LNCs enhance the delivery and uptake of drug; structure-guided redesign of existing agents lowers toxicity and delays resistance; and the discovery of natural antimicrobial peptides extend the selectivity of antifungal drugs. The application of currently available resources and the development of technology will be important in our fight against future fungal infections and increasing drug resistance.
